# Self-Management of Medications During Sick Days for Chronic Conditions: A Scoping Review

**DOI:** 10.3390/medicina61101742

**Published:** 2025-09-25

**Authors:** Mimi Truong, Kamal Sud, Connie Van, Wubshet Tesfaye, Vani Nayak, Ronald L. Castelino

**Affiliations:** 1 School of Pharmacy, Faculty of Medicine and Health, The University of Sydney, Sydney 2006, Australiavnay0219@uni.sydney.edu.au (V.N.); ronald.castelino@sydney.edu.au (R.L.C.); 2 Pharmacy Department, Blacktown Hospital, Western Sydney Local Health District, Blacktown 2148, Australia; 3 Nepean Kidney Research Centre, Department of Renal Medicine, Nepean Hospital, Nepean and Blue Mountains Local Health District, Sydney 2747, Australia; kamal.sud@health.nsw.gov.au; 4 Sydney Medical School, Faculty of Medicine and Health, The University of Sydney, Sydney 2006, Australia; 5 School of Pharmacy and Pharmaceutical Sciences, Faculty of Health, Medicine and Behavioural Sciences, The University of Queensland, Brisbane 4072, Australia; w.tesfaye@uq.edu.au

**Keywords:** chronic disease, medication management, medicine information, patient education, patient empowerment

## Abstract

*Background and Objectives*: Sick-day medication guidance involves patients self-adjusting medications during sick days to prevent adverse events and minimise exacerbation of their disease states. This review aimed to summarise and synthesise all sick-day interventions provided by healthcare professionals (HCPs) for patients with chronic illnesses, including diabetes, cardiovascular disease, chronic kidney disease (CKD), adrenal insufficiency (AI), rheumatoid arthritis, chronic obstructive pulmonary disease (COPD), and asthma. *Materials and Methods*: A search of Embase, Medline, International Pharmaceutical Abstract, Scopus, Google Scholar, and the grey literature was conducted until July 2025. The review followed the methodological framework according to the Preferred Reporting Items for Systematic Reviews and Meta-Analyses extension for scoping reviews. Data were extracted using a modified TIDieR checklist, and the findings were summarised descriptively and presented thematically. *Results*: The search included 6932 documents, and 97 met the inclusion criteria: 57 published guidelines/education resources and 40 pieces of original research. Seventy-four interventions were identified for diabetes (18), asthma (32), AI (8), CKD (6), AKI prevention (4), COPD (4), and mixed conditions (2). The most common type of intervention was written information (action plans and information sheets), with education mostly provided by multidisciplinary teams. Novel interventions included 24h phone support and an educational mobile application. Participants showed interest in sick-day interventions and HCPs viewed these interventions as effective, important, and easy to provide. However, interventions did not always translate to improved clinical outcomes, with studies reporting mixed outcomes regarding healthcare utilisation. Nonetheless, some interventions showed improved patient knowledge and satisfaction with care. *Conclusions*: Multiple interventions are available for asthma and diabetes, with fewer targeting CKD or acute kidney injury (AKI) prevention. While demand for these interventions from consumers and HCPs is high, implementation challenges and inconsistent benefits remain. Further primary research is needed to clarify which intervention features are most effective in yielding meaningful clinical outcomes.

## 1. Introduction

Chronic diseases are widespread, affecting approximately 50% of the Australian population [1]. Although medications are an integral part of chronic disease management, there are risks associated with their use, including drug-related problems (DRPs) and potential drug interactions. Studies have shown that 2.5% of all hospital admissions are medication-related and potentially preventable [2], showing the importance of facilitating safe medication use to prevent DRPs. One area of concern is the use of medications during episodes of acute illness, where prescribed doses of medications can cause harm or be ineffective. During acute volume-depleting illnesses, pharmacokinetic and pharmacodynamic changes affect medications used to manage diabetes, cardiovascular disease, chronic kidney disease (CKD), asthma, and adrenal insufficiency, which can result in inadequately controlled symptoms and DRPs such as hypotension, diabetic ketoacidosis, hypoglycaemia, and acute kidney injury (AKI) [3].

To address these issues, various organisations have recommended providing patients affected by some of these conditions with sick-day medication guidance (SDMG) containing advice on how to adjust medications, including temporarily withholding some medications during an episode of acute illness or increasing therapy. This advice could come in the form of written information or verbal counselling, allowing patients to self-manage their medications appropriately, reducing their risk of exacerbation of symptoms (i.e., in people with asthma or adrenal insufficiency) or DRPs, such as those with diabetes, cardiovascular disease, or CKD. Overall, the implementation of SDMG has been poor. For patients with asthma, only 71% of people have a written asthma action plan [4]. Similarly, one study showed that only 15% of HCPs provide SDMG regarding cardiovascular, diabetes, and kidney disease medications [5]. It has been found that the failure to adjust medications during sick-day events has been a potential cause of adverse drug events including hospitalisations [6].

Previous reviews on sick-day medication management have found an imbalance between the number of documents with SDMG and the interventions that have attempted to implement these guidelines [7]. Lack of SDMG implementation may be due to variability between organisations’ guidelines and weaker evidence bases for SDMG since most of them are based on expert consensus [5]. Previous reviews have narrowed their focus to SDMG for medications used to manage diabetes, kidney disease, and cardiovascular disease [5,7]; however, it is known that other medications like corticosteroids used to manage adrenal insufficiency also require SDMG [3]. To date, no previous reviews have attempted to capture and summarise sick-day interventions available for all chronic conditions, or the impact of these interventions on patient outcomes. By synthesising interventions across multiple chronic conditions, this review will clarify whether challenges with implementation are disease-specific or reflect systemic issues with SDMG conceptualisation and integration. Furthermore, this review will identify the common characteristics of effective interventions, transferable strategies across diseases, and critical gaps in current practice and research. Hence, the aim of this scoping review is to summarise and synthesise sick-day guidance interventions provided by healthcare professionals (HCPs) in patients with all chronic illnesses, including but not limited to diabetes, cardiovascular disease, CKD, adrenal insufficiency, rheumatoid arthritis, chronic obstructive pulmonary disease, and asthma, and where available, to summarise the outcomes of providing such guidance.

## 2. Materials and Methods

**Figure 1:**
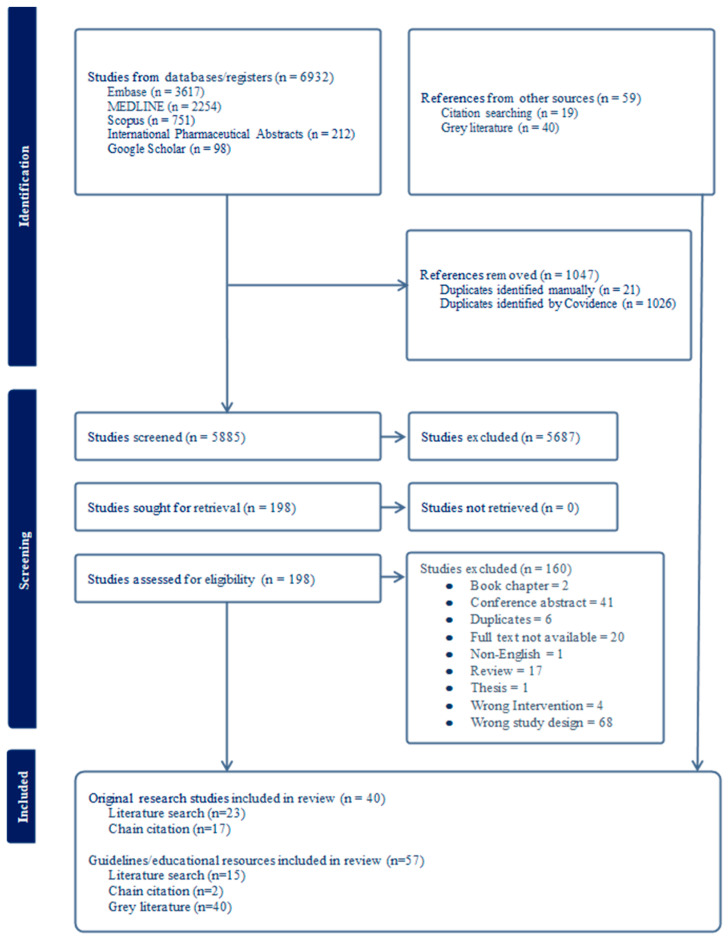
Flow diagram of Preferred Reporting Items for Systematic Reviews and Meta Analyses Statement for Scoping Reviews.

A scoping review was conducted using the methodological framework as outlined by Arksey and O’Malley [8], developed by Levac et al. [9]. The framework includes identifying the research question, identifying relevant studies, selecting studies, charting the data, collating, summarising, and reporting results. This review also integrated updated methodological guidance from Peters et al. [10]. Additionally, this scoping review was reported according to the Preferred Reporting Items for Systematic Reviews and Meta-Analyses Statement for Scoping Reviews (PRISMA-ScR) (Figure 1) and Guidelines. The PRISMA-ScR checklist is available in Supplementary S1. A scoping review was chosen given that the concept of ‘sick-day management’ is contemporary and the associated literature is heterogenous and therefore best mapped using this method. A protocol for this scoping review was not registered.

### 2.1. Identifying the Research Question

The research question was developed using the population, concept, and context by JBI. This review sought to determine what ‘sick-day medication management’ interventions are being provided by healthcare professionals (concept) for people with chronic diseases (population), across all levels of care (context), and what is known about their outcomes. For the purpose of this review, ‘sick-day medication management’ was defined as any verbal or written information provided to patients to temporarily change their medication(s) themselves during acute illness [5]. This review was conducted to expand the findings of Watson et al.’s [7] and Duong et al.’s [5] reviews, both of which examined sick-day medication management in the context of diabetes, cardiovascular disease, and kidney disease—but did not account for sick-day management for other chronic diseases.

### 2.2. Search Strategy and Eligibility Criteria

A search strategy was created around the research question, with the key concepts identified as a “sick day”, “medication management” and the chronic disease of interest, which was initially searched as “chronic disease” before being narrowed to specific diseases including diabetes, cardiovascular disease, diabetes mellitus, chronic obstructive pulmonary disease (COPD), asthma, rheumatoid arthritis, CKD, and adrenal insufficiency (Supplementary S2). The peer-reviewed literature was included if it discussed the implementation of an intervention, whereby HCPs provided some form of sick-day medication management advice. This included primary research articles and guidelines or educational resources. The following types of publications were excluded: clinical trial registrations, case reports, conference abstracts. The search strategy was created by M.T., discussed with another investigator R.L.C., and further refined by a librarian.

A search was conducted in Embase, Medline, International Pharmaceutical Abstract (IPA), Scopus, and Google Scholar, with the most recent search executed in July 2025, by M.T. and R.L.C. The peer-reviewed literature was included if it described the implementation of an intervention aimed at medication management during sick days. A grey literature search was also conducted in Google Scholar by searching the same key concepts. A Google search of the first three pages was also conducted, seeking organisational documents and resources with the key words “sick day” and its synonyms. All of the literature selected for this study was written in English with no limitations on publication date to increase the yield of results and reduce the risk of publication bias [11]. We excluded non-English publications due to accessibility issues, since limited resources were available for translation. This review excluded documents that were position statements, commentaries, and case studies, not written in English, or discussed a “sick day” in the context of occupational sick leave. The full electronic search strategy for each database has been reported in Supplementary S3. Forward and backward citation chaining was also performed for publications included for full-text review to identify additional studies that were not captured in the initial search.

### 2.3. Study Selection, Data Extraction, and Analysis

All studies identified from the initial search were exported to Covidence [12], a screening and data extraction tool for systematic reviews. After removing duplicates, titles and abstracts were independently screened by two researchers (M.T. and R.L.C.) against the eligibility criteria. One author (M.T.) conducted full-text screening, which was validated by a second researcher (R.L.C.). Discrepancies regarding inclusion were resolved through discussion.

All data were extracted to a charting table using Microsoft Excel (Version 16.87), which included the author, year, country of publication, article type, intended audience, participant characteristics, disease state, outcomes (if available), source of evidence, and some aspects of the Template for Intervention Description and Replication (TIDieR) checklist [13]. The charting table was refined iteratively after piloting with five publications. This charting table was then summarised, with definitions of the data items provided in Supplementary S3. Findings were summarised descriptively and presented thematically.

## 3. Results

Overall, 6932 publications were retrieved from biomedical database searches. After removing 1047 duplicates, 5885 studies were screened, and 5687 were determined to be irrelevant. Subsequently, 198 studies were assessed for eligibility, of which 38 were included. Forward and backward citation chaining of these eligible studies was conducted, and the grey literature was also searched. This process identified an additional 59 sources. In total, 97 works related to sick-day medication advice interventions were identified; of these, 40 were original research articles and 57 were guidelines or educational resources (Figure 1).

### 3.1. Document Characteristics—Original Research

The characteristics of the original research documents are summarised in Table 1. Most research was conducted in North America (*n* = 24, 35%), Europe (*n* = 13, 33%), and Oceania (*n* = 10, 25%). The interventions addressed various conditions including asthma (*n* = 19, 48%) [14,15,16,17,18,19,20,21,22,23,24,25,26,27,28,29,30,31,32,33,34], COPD (*n* = 6, 15%) [24,34,35,36,37,38,39,40], CKD (*n* = 2, 5%) [41,42], AKI prevention (*n* = 4, 10%) [39,43,44,45], type 1 diabetes mellitus (*n* = 6, 15%) [34,46,47,48,49,50,51], type 2 diabetes mellitus (*n* = 1, 3%) [34,52], and a mixture of these conditions (*n* = 2, 5%) [40,43,44]. All articles described an intervention, with the research methods including randomised controlled trials (*n* = 23, 58%), uncontrolled trials (*n* = 8, 20%), and retrospective cohort studies (*n* = 3, 8%). Included populations were mostly adults, with limited studies in asthma and type 1 diabetes mellitus including children and young adults.

### 3.2. Sick-Day Definition

Definitions of sick days or exacerbations requiring medication adjustments were provided in most research articles, defined by either symptoms and or functional/biochemical tests. Eleven articles did not explicitly report what they considered to be a ‘sick day’ [15,18,19,20,25,26,28,45,47,52,53].

“Sick days” or “exacerbations” were most clearly defined in asthma interventions—normally as peak expiratory flow, ranging between studies from <60 to 80% of personal best peak expiratory flow [14,17,21,22,23,24,27,29,30,31,33]. Some articles also defined sick days using symptoms including ‘waking at night with a cough or wheeze’, ‘persistent cough’, ‘common cold symptoms’ [16,21,22,31,32,33], or needing to bronchodilator more often [33]. In contrast, in COPD, exacerbations were mostly defined by symptoms including an ‘increased cough’, changes in sputum (colour, purulence, volume), ‘dyspnoea’, ‘wheezing’, ‘reduced energy’, ‘poor sleep’, ’loss of appetite’, and so on [35,37,38,39,40].

Insulin adjustment in the context of type 1 diabetes mellitus was clearly defined, often only in the presence of elevated blood or urine ketones (>1.5 mmol/L) [49] and/or blood glucose levels [46,48,51]. Symptoms used to define sick days included ‘fever’, ‘chills’, ‘nausea’, ‘vomiting’, ‘inability to tolerate food’, or ‘feeling unwell’ [46,48,50,51].

For medications used in cardiovascular and kidney disease, sick days were defined by volume-depleting symptoms including ‘excessive vomiting’, ‘diarrhoea’, ‘fever’, ‘sweats’, ‘shaking’, ‘poor fluid intake’, ‘thirst’, ‘weight loss’, ‘light-headedness’, and ‘fatigue’ [41,42,43,44].

### 3.3. Interventions Addressing Sick-Day Medication Management

A summary of the interventions used to support sick-day medication management is given in Table 2. These studies employed a variety of interventions to address sick-day management, including education (*n* = 6, 15%), written information (*n* = 4, 10%), phone support services (*n* = 2, 5%), and ongoing support (defined as regular, scheduled contact with patients that was not related to study follow-up data collection). Interventions were often delivered as a combination of these methods, with the most common being education with written information (*n* = 16, 40%), then education, written information, and ongoing support (*n* = 7, 18%). Written information was mostly in the form of action/management plans and information booklets.

Sick-day interventions were almost equally delivered in community settings (*n* = 18, 45%), such as medical centres, clinics, and pharmacies, and in hospital-based settings (*n* = 17, 43%) like outpatient clinics. Fewer interventions were delivered across multiple settings (*n* = 5, 13%). Most interventions were delivered individually to the participant (*n* = 32, 80%), and they were often tailored (*n* = 28, 70%). Of the interventions that were delivered by HCPs (*n* = 38), the most common providers were multidisciplinary teams (*n* = 12, 32%), followed by nurses (*n* = 9, 24%), pharmacists (*n* = 6, 16%), and doctors (*n* = 4, 11%).

Some interventions included novel features, such as the use of technology (e.g., mobile applications), or design elements aimed at enhancing convenience (e.g., card-sized materials for portability, magnetic materials for visibility). The contents of these interventions were described in-depth for most interventions (*n* = 35). The most common components included instructions to enable self-adjustment of prescription medications (*n* = 33, 94%), prompts to contact a primary HCP (*n* = 28, 80%), a definition of a ‘sick day’ to prompt medication changes (*n* = 19, 48%), advice to increase self-monitoring (*n* = 11, 31%), non-pharmacological/lifestyle advice (*n* = 11, 31%), and prompts to contact emergency services (*n* = 6, 17%).

### 3.4. Patient Engagement and Capability 

The interventional studies showed varying levels of engagement with sick-day interventions. In one study, 58% (*n* = 54) of participants recalled receiving the intervention [43], and in other studies, 65% (*n* = 13) [51] and 90.6% (*n* = 551) [34]. A separate study noted that the sick-day resource was one of the most accessed components of a self-navigated, consumer-focused resource toolkit—indicating consumer interest and engagement [47].

Patient implementation of sick-day interventions varied across studies, including 43% (*n* = 3) [31], 62% (*n* = 58) [43], 60% (*n* = 33) [16], and 61.4% (*n* = 373) [34]. Another aspect examined was participants’ ability to adhere to the intervention protocol. This included two main components: (1) ability to identify sick days requiring medication changes appropriately, and (2) following the recommended actions per the intervention correctly. Success in identifying true sick days varied substantially from 51.4% (*n* = 19) [41] to 92% (*n* = 606) [38] and up to 98% (*n* = 82) [49].

Adherence to medication changes as per the intervention was also varied and determined at the end of the study. For instance, in a randomised controlled trial focused on withholding medications in CKD, only 49.2% (*n* = 14) followed the protocol correctly [41], with some errors including stopping the wrong medication [41]. However, in another intervention, post-education interviews with CKD patients showed that 90% (*n* = 52) of participants correctly identified all medications requiring withholding [43]. Similarly, a usability testing study for an educational tool found that 83% (*n* = 10) easily completed at least 90% of their tasks—which involved identifying sick-day symptoms and knowing which medications to withhold [15].

However, in asthma interventions—which often required increased medication use, such as dose escalation or the addition of other medications—adherence rates appeared to be higher. For example, full compliance with the protocol was reported in 86% of exacerbations (*n* = 31) in one study [33], 62% (*n* = 87) in another [29], and 45% (*n* = 9) in a third study [27].

### 3.5. Patient Experiences and Perceptions of Sick-Day Interventions

Studies that included patient perspectives indicated an overall positive attitude towards sick-day interventions [15,16,27,32,33,34,35,41,42,43,47,51]. In certain studies, 38% (*n* = 35) and 90% (*n* = 26) of participants reported a positive or very positive reaction to the intervention [35,43]. Other studies described higher satisfaction with care that involved interventions compared to standard care [15,33,52].

Reasons explaining satisfaction included ease of use—especially for interventions involving technology like apps with videos [47], tablet-based education modules [42], and asthma action plans [16]. Perceived benefits from interventions included enhanced knowledge about the condition [15,16], better disease control [16,35], improved adherence to medications [32], enhanced understanding of adverse events [42], improved self-confidence [32], improved ability to self-manage sick days [43], and support for carers in managing sick days [32,47].

These tools were especially valued in situations where contacting HCPs was difficult [51]: “It worked brilliantly for us and yeah, I think prevented us from having to get in touch with HCPs who were busy at the time, and it kept X well” [47].

The long-term engagement with these tools is evidenced by many participants wanting to use interventions post-study [41,52]; for instance, in some studies, 45% (*n* = 42) [43] and 88% (*n* = 29) still knew where to access the intervention, and 39% (*n* = 13) continued to use it post-study [27]. Engagement was also indicated by participants showing a strong willingness to share these resources with others [42]. In one study, 68.5% (*n* = 798) agreed or strongly agreed that they would recommend the intervention to others [47].

### 3.6. Outcomes Measured

The outcomes measured were categorised into patient-centred outcomes and healthcare system utilisation. Patient-centred outcomes included disease management, knowledge about the disease, adherence, and quality of life. Healthcare system utilisation measures included general practice (GP) presentations, emergency department visits, hospitalisations, and readmission rates.

#### 3.6.1. Patient-Centred Outcomes

For asthma interventions, disease management was measured using both subjective and objective measures. Subjective measures included self-rated assessments to describe symptoms such as simplistic self-rating scales [19,21,35] and other standardised scales like the “Asthma Bother Scale” [20] and “Asthma Control Test” [26,28]. Objective measures included lung function measures, such as FEV1 or peak expiratory flow [19,23,27,28,31,33,35,37,40], or reported symptoms, including shortness of breath and wheezing [16,19,21,25,28,30,32]. To assess disease management, some studies measured the incidence rate of the targeted adverse events they were trying to prevent, for instance, number of exacerbations or sick days [19,32,33,37,38,41], AKI episodes [41], or diabetic ketoacidosis [48]. Other conditions also reported changes in biomarkers like HbA1c [46,48,49] and eGFR [41,45].

Many interventions aimed to determine changes in knowledge by assessing knowledge pre- and post-intervention [18,19,26,28,31,46]. Various tools were used to assess knowledge, including standardised ones like the “Knowledge of asthma and asthma medicine” questionnaire [26] and “Asthma: Fact and Fiction” [20], or researcher-developed ones [18,19,28,46,50,52]. The contents of these assessments varied per study, but many included knowledge about the condition [19,20,26], treatment during exacerbations/sick days [19,20,28,31,50,52], side effects of medications [26], and non-pharmacological management strategies [26]. Multiple interventions showed improved knowledge post-intervention [18,19,20,28,31,50,52]. Notably, one study assessed changes in participants’ perceived information needs rather than objective knowledge, reporting an increase in the proportion of participants who indicated that they “know enough” rather than “want to know more” post-intervention [15].

Five studies reported adherence as an outcome using various tools. These included externally validated instruments such as the Morisky Medication Adherence Scale (MMAS) [26], Malaysia Medication Adherence Scale (MMAS) [28], and Adherence to Refills and Medications Scale (ARMS) [45]. Other studies measured adherence based on how often prescribed medications were dispensed [24] or the number of doses taken [17]. Most studies showed improved adherence [17,24,26,28], though one showed no significant changes [45].

Quality of life was measured using externally validated tools like Hyland’s score [27], St George’s Respiratory Questionnaire (SGRQ) [35,37,39,40], its variants [29], or other unnamed validated tools [18]. Quality of life was eight points higher in the self-management group than the traditional treatment group [29]. Another study found an improvement, but that was not statistically significant [40]. Some studies reported no differences in quality-of-life score between groups [27,35,39], while others observed improvements within specific domains such as ‘activity’ and ‘impact’ [37] and ‘symptoms’ [18].

#### 3.6.2. Healthcare System Utilisation

Healthcare system utilisation was assessed via the number of GP visits—both routine and emergency—and hospital encounters, including outpatient visits, emergency department presentation, and readmissions. Certain interventions were associated with reduced routine GP visits [19,22,37,38]. For example, one study reported that each patient who received the intervention had 1.79 fewer unscheduled GP contacts compared to the control group [37]. However, other studies found no significant differences in the number of GP visits between intervention and control groups [37,39,40].

Emergency department presentations were also evaluated. Some studies reported reductions in emergency department presentations [16,19,34,38,46]. Notably, one study showed a significant decrease from 18 to 6 (*p* = 0.023) emergency department visits [16]. In contrast, other studies found no differences in emergency department presentation rates between groups [39,40,49].

Hospitalisation rates were also analysed. One study found that a higher proportion of patients in the intervention group required hospitalisations compared to the control group (12% vs. 3%) but this was not significant [14]. Other studies reported no difference in hospitalisation rates between groups [19,40]. Some studies showed fewer hospital admissions in the intervention group [16,32,38] and reductions in hospital readmission rates [19,21,25].

### 3.7. Healthcare Professional Experiences and Perceptions of Sick-Day Interventions

Only 13% (*n* = 5) of studies included perspectives from HCPs [27,34,35,44,47]. Overall, sick-day interventions were perceived positively by HCPs and were considered helpful in managing patients’ condition. In one study, 84% (*n* = 149) agreed or strongly agreed that the intervention helped manage the patient’s condition [47]. Another study highlighted the usefulness in recommending additional monitoring [27], and how interventions can reduce confusion in patients: “Patients feel reassured as [they] often become confused about which meds should take if unwell” [44]. HCPs also noted that the interventions raised awareness about the risks of continuing medications while dehydrated: “Most of the patients I gave the cards to had no idea that there could be an issue with serious consequences if they carried on taking these medicines when dehydrated” [44].

One study found that 79% (*n* = 62) of HCPs considered sick-day action plans safe and effective [34]. This belief was reflected in practice, where 71% (*n* = 55) of participants supplied the intervention to patients [44], and in another study in which 55.6% (*n* = 99) reported that they very frequently provided patients with sick-day advice [47]. The sustained use of these tools beyond the study period further reflects positive attitudes, with some HCPs expressing a willingness to continue using them [35].

Additionally, 94% (*n* = 19) of HCPs reported no difficulty explaining the action plan to their patients [35]. Of the 6% (*n* = 1) who experienced challenges, the primary issue was the additional time required for explanation [35].

HCPs also provided suggestions for how to improve the usability of sick-day interventions. These included clarifying the level of illness that warrants using the guidance (“Perhaps more clarity on level of sickness/diarrhoea which needs to use these rules” [44]), and enhancing accessibility, such as improving font size for patients with visual impairments and simplifying the content for those with limited health or general literacy [34].

### 3.8. Document Characteristics—Guidelines and Educational Resources

The characteristics of guidelines and other resource documents are summarised in Table 3. In total, *n* = 57 documents and educational resources were identified. These were mostly from North America (*n*= 18, 32%), Europe (*n* = 16, 28%), and Oceania (*n* = 15, 26%). The resources addressed various conditions, including asthma (*n* = 14, 25%) [54,55,56,57,58,59,60,61,62,63,64,65,66,67], adrenal insufficiency (*n *= 9, 16%) [68,69,70,71,72,73,74,75,76], type 2 diabetes mellitus (*n* = 9, 16%) [77,78,79,80,81,82,83,84,85], type 1 diabetes mellitus (*n* = 5, 9%) [86,87,88,89,90], type 1 and type 2 diabetes (*n* = 8, 14%) [91,92,93,94], COPD (*n* = 2, 4%) [95,96], CKD (*n* = 4, 7%) [97,98,99,100], heart failure (*n *= 3, 5%) [101,102,103], and AKI prevention (*n* = 3, 5%) [104,105,106]. Of the 57 resources, 34 (60%) included written information for patients, mostly in the form of action plans (*n* = 19, 56%) and patient information sheets (*n* = 9, 26%). Of these resources, *n* = 16 (47%) contained tailored advice about treatment.

## 4. Discussion

Self-management in chronic disease requires individuals to actively manage their health to prevent disease progression and maintain quality of life. Key self-management skills include understanding the condition and having the ability to respond to early warning signs and symptom changes [107]. While each chronic condition presents unique features, common challenges include managing complex medication regimens and monitoring physical indicators of disease progression [108]. This scoping review adds to the existing literature by summarising interventions that support medication self-management during sick days or exacerbations across conditions including asthma, cardiovascular disease, COPD, diabetes, heart failure, adrenal insufficiency, AKI prevention, and CKD. To our knowledge, this is the first review to consolidate all sick-day interventions across multiple conditions, whereas previous reviews have focused on individual conditions in isolation [35,109,110,111]. This review described the characteristics of the interventions, reported clinical outcomes, and perspectives of both patients and HCPs. By examining a wide range of chronic conditions, this review was able to identify shared challenges and potentially transferrable strategies, which may inform the development of more effective, integrated self-management approaches during acute illness.

### 4.1. Interventions Available

Overall, 97 interventions aimed at enabling patients to manage sick days were identified in this review, with most resources available for asthma and diabetes, and fewer for COPD, CKD, AKI prevention, and heart failure. Interventions were usually in the form of written materials, including action plans and patient information leaflets; these were provided in conjunction with patient education, which is highly recommended by guidelines and preferred by HCPs [112,113]. Interestingly, interventions from original research articles were more frequently tailored (70%) compared to resources available from guidelines or the grey literature (47%), suggesting that non-tailored information is currently more accessible. Tailored interventions are recommended [114], as generic materials may contain irrelevant and inappropriate information given the individual patient circumstances. Prior studies have shown that individuals will often adjust their plans according to their personal beliefs and experiences with the disease, highlighting the importance of patient involvement when tailoring these interventions [115]. Sick-day interventions were provided in both community and hospital settings, mostly by multidisciplinary healthcare teams. Notably, no studies reported sharing interventions with other HCPs, such as GPs, through digital health record systems like My Health Record [116]. Enhancing communication through digital health record systems could improve the consistency in messaging between HCPs, which is a barrier to implementation [113].

Most interventions were only delivered once during the study period; however, given the complexity of medication management during sick days, repeated delivery may be more beneficial and should be explored in future research. Education and action plans were often delivered by non-prescribers, including pharmacists and specialised practice nursers, highlighting their capability to provide and support patient education. However, barriers to real-life application show that non-prescribing HCPs are concerned about overstepping legal and professional boundaries [113]. This highlights the need to develop clear protocols and legal guidelines to support better translation in practice.

Reported outcomes varied, with some studies reporting increased healthcare utilisation, such as emergency department visits, while others observed reductions. This reflects a key challenge in evaluating sick-day interventions, as these resources may prompt patients to seek urgent care more frequently. Nevertheless, a consistent benefit observed was improved patient knowledge and greater satisfaction with care, which may support broader aspects of chronic disease self-management.

### 4.2. Benefits and Practical Applications

Participants in the included studies generally held positive attitudes towards sick-day interventions, viewing them as tools that enhanced their ability to self-manage medications during periods of acute illness. However, the actual implementation of interventions varied significantly between studies. This variation could be due to infrequent sick days during the study period, or practical challenges related to health literacy [117,118]. For instance, participants are required to identify when a sick day requires action and implement the appropriate action. These problem-solving and decision-making skills are core self-management competencies, as described by Lorig et al. [119], and demand a high level of health literacy. Additionally, participants’ actions could be influenced by their perception of risk in applying the advice. For example, managing an asthma exacerbation requires an increased use or dosage of a current medication, or the addition of therapy, which may be perceived as less risky than withholding medications, as is required for sick days in CKD. This may explain why adherence sick-day interventions for asthma is higher than for CKD. However, this perceived risk has not been formally explored in qualitative studies.

Implementation challenges have also been highlighted in other reviews on sick-day interventions [35,109,110,111] and may explain why some patients prefer to contact an HCP before making medication changes [120,121]. Development of patient–provider partnerships and action planning are again considered core self-management skills [119].

These challenges are exacerbated in culturally and linguistically diverse (CALD) people, who face additional language barriers, and those with limited health literacy. Furthermore, many studies excluded people from CALD backgrounds, despite guidelines recognising them as a priority group [122], thus limiting the generalisability and equity of sick-day interventions.

Overall, the challenges with practical application identified in this review reinforce the need for interventions to incorporate all facets of self-management skills, including problem solving, decision-making, resource utilisation, formation of patient–provider partnership, action planning, and self-tailoring [119]. Embedding these components is likely to ensure that self-management programs are effective and achieve optimal patient outcomes [119]. Interventions should be tailored and delivered in ways that suit an individual’s health literacy levels, cultural contexts, and communication needs.

This review found considerable variation in how “sick days”, or exacerbations, were defined within each condition. In some studies, sick days were defined by symptoms alone, while others incorporated measurable indicators like functional or biochemical tests. This lack of standardisation may contribute to patient confusion and limit the effectiveness of sick-day guidance—particularly in conditions like CKD and adrenal insufficiency, where definitions often rely only on symptoms or specific scenarios. Even clinical guidelines that recommend these sick-day interventions have differences in their definitions of “sick days”, which likely explains why definitions within interventions are so varied. Establishing standardised definitions of “sick days” [5,123] is essential to improve consistency across resources and enhance patient understanding.

### 4.3. Potential Risks and Harms

A concern in implementing sick-day interventions is the potential risk of harm. In this review, many participants showed non-adherence to the study protocol/intervention—for instance, failing to recommence therapy after recovering from the illness, and failing to adjust medication appropriately during the illness, for example, underdosing themselves during an asthma flare. This occurred despite some interventions undergoing usability testing beforehand, highlighting the challenges of simulated versus real-world application of these interventions.

Some HCPs also have concerns about withholding medications due to the risk of potentially exacerbating the underlying condition, particularly when withholding diuretics in heart failure [113]. Whilst many sick-day interventions for asthma have shown benefits in reducing hospital admissions and decreasing exacerbation severity, the same benefits have not been determined for sick-day plans in CKD and type 2 diabetes [113], with most recommendations based on expert consensus [5,7]. The lack of primary evidence further contributes towards HCPs’ uncertainty about applying interventions in practice.

### 4.4. Strengths and Limitations

The strengths of this study are that the search strategy was comprehensive and yielded a high volume of articles. Additionally, as it included all chronic conditions, it provided a broad understanding of intervention implementation and common challenges. However, there are some limitations to be acknowledged for this review. Firstly, scoping reviews aim to map the breadth of the literature and identify gaps in knowledge, rather than synthesise evidence or assess effectiveness as in systematic reviews with meta-analyses. Secondly, the grey literature search was conducted via Google Scholar and Google within Australia. Given that search algorithms are influenced by the user’s location, this may have limited the results overall, with a bias towards resources published in Oceania. Additionally, risk-of-bias and quality appraisals were not conducted. Another limitation is that no eligible articles were identified regarding the use of corticosteroids for rheumatoid arthritis. This highlights a specific research gap within the broader literature on chronic condition interventions, which warrants further investigation. Finally, the heterogenicity of interventions and outcomes across studies made their direct comparison challenging—future reviews that focus on standardised outcome measures may yield more meaningful comparisons. 

## 5. Conclusions

This scoping review identified a variety of sick-day medication guidance and interventions for chronic conditions, mostly asthma and diabetes, and fewer for CKD and AKI prevention. Written information was the most common type of intervention, primarily sourced from guidelines and educational resources, rather than primary research articles. Although reported outcomes varied between interventions, most studies highlighted that patients and HCPs had positive attitudes toward them, but they experienced challenges with real-world application. These challenges can be addressed by ensuring that interventions incorporate all core components of self-management and that they are tailored and delivered in a way that meets the individual needs of the patient, as recommended by guidelines. Additionally, given the conflicting outcome data from these interventions, more primary research is required to clarify which intervention features are most effective in achieving meaningful clinical outcomes.

## Figures and Tables

**Table 1:** Summary of study characteristics (*n *= 40).

Characteristic	Number of Documents *n* (%)
Study design	
Descriptive	1 (3%)
Development and usability study	1 (3%)
Evaluation	2 (5%)
Mixed methods	1 (3%)
Prospective cohort study	1 (3%)
Retrospective cohort study	3 (8%)
Randomised controlled trial	23 (58%)
Uncontrolled trial	8 (20%)
Publication year	
Before 2000	18 (45%)
2000–2009	7 (18%)
2010–2019	4 (10%)
2019–2025	11 (28%)
Study age group	
Children (<18 years old)	9 (23%)
Children and adults	6 (15%)
Adults	25 (63%)
Disease state(s)	
Asthma	19 (48%)
Acute kidney injury prevention	4 (10%)
Chronic kidney disease	2 (5%)
Chronic obstructive pulmonary disease	6 (15%)
Type 1 diabetes mellitus	6 (15%)
Type 2 diabetes mellitus	1 (3%)
Mixture of chronic conditions, i.e., cardiovascular disease	2 (5%)
Region of origin	
Asia	3 (8%)
Europe	13 (33%)
North America	14 (35%)
Oceania	10 (25%)

**Table 2:** Characteristics of interventions to manage medications during sick days (*n* = 40).

Author(s), Year	Condition	Intervention Type	Intervention Name	Intervention Description	Setting (Format)	Facilitator	How Often Intervention Was Delivered by Facilitator	Tailoring to Participant
Charlton et al., 1994 [14]	Asthma	Education, written information, ongoing support	Patient education program and self-management plan	Interview to determine history of the asthma, provoking factors, and current symptoms. Medications were adjusted if needed. Brief education and provision of tailored self-management plan. Follow-up review with either GP nurse or GP every 3 months.	Hospital—outpatient clinic, community—medical centre (individual)	Education—asthma nurse, reinforcement —GP nurse, GP	Once at baseline	Management as per personal PEF
Farrell et al., 2011 [49]	T1DM	Phone support service	24 h mobile phone support service	Participants educated about testing for ketones when unwell and to contact the 24 h mobile phone support in the presence of ketones.	Hospital—outpatient clinic (individual)	N/A	Discussion at every clinic visit	N/A
Fink et al., 2022 [41]	CKD	Written information	NHS Scotland’s card-sized “Medicine Sick Day Rules” with weekly remote monitoring	Participants followed instructions on the card to withhold medications for up to 48 h while sick and recommence once well again. Additional pamphlet describing a sick-day event and providing further instructions also given.	Community—medical centre (individual)	N/A	N/A	N/A
Madge et al., 1997 [25]	Asthma	Education, written information, ongoing support	Education program and “Going home with asthma” booklet, card-sized management plan	Education and resources given to participants. Telephone support of nurse available throughout study.	Hospital—inpatient (individual)	Education—asthma nurse	Education over 2 sessions	Individualised plan
Vicary et al., 2020 [43]	AKI prevention	Education and written information	Education program and sick-day guidance sheet	Education and resource given to participants.	Community—pharmacy (individual)	Education—pharmacist	Once at baseline	N/A
Wilson et al., 1993 [20]	Asthma	Education	Education program with workbook	Either group education with group discussion and group exercises, or individual education (content dependent on patient’s needs as per nurse educator’s assessment). Both arms received the workbook for self-study and could call the educator with any questions.	Community—medical centre (individual or group)	Education—nurse educator	For group education: 4 times, for individual education: 3–5 times	Individual education individualised
Cote et al., 2001 [23]	Asthma	Education and written information	Self-action plan with or without structured education	All participants received a self-action plan based on symptoms or PEF. Some participants also received structured education.	Hospital (individual or group)	N/A	Education at baseline, 6 months	Management as per predicted PEF or personal PEF
Kime et al., 2023 [47]	T1DM	Education	“DigiBete” mobile phone application	Participants self-navigated 200 educational videos on topics including “My Sick Day Rules”. The application can be used to receive direct communication from diabetes team.	Hospital (individual)	Not applicable	N/A	N/A
Kovacevic et al., 2018 [26]	Asthma	Education and written information	Structured education and asthma action plan	Participants were given structured education and an asthma action plan.	Community—pharmacy (individual)	Pharmacist	Education at baseline and 3 months (optional)	N/A
Thoonen et al., 2002 [15]	Asthma	Education	Education program	Education was provided and participant completed feedback form on whether they received information during the previous session. Gaps in knowledge were filled in subsequent education sessions.	Community—medical centre (individual)	GP	Education over 4 sessions	Education tailored according to participant’s needs
Bowman et al., 2020 [42]	CKD	Education	Tablet-based mobile educational tool	Participants were given a tablet and listened to auditory explanations with complimentary graphics, i.e., photographs of medications to be withheld during acute illness. They were then given a clinical scenario and questions regarding the hypothetical patient. They were then assessed and had to choose which medications the patient in the clinical scenario had to withhold.	Hospital—outpatient (individual)	Not applicable	Once at baseline	N/A
Klein et al., 1997 [31]	Asthma	Education and written information	Group education and written guidelines to self-adjust medications	Participants attended group-education sessions and received written guidelines to self-adjust medication according to PEF values/symptoms.	Hospital—outpatient (group)	Nurse	Education over 4 sessions	Management as per personal PEF
Morrison et al., 2014 [44]	Not specified	Written information	“Medicine Sick Day Rules”	HCPs provided cards to participants who were taking at-risk medications.	Community—pharmacy, medical centre, hospital (individual)	GP, pharmacist	Whenever medication was supplied	N/A
Kado et al., 2022 [52]	T2DM	Education and written information	A5 sick-day card	Education was provided and sick-day card with recommended medication adjustments was inserted into the participant’s medication handbook. Recommended adjustments needed to be signed off by GP at next visit and cited by pharmacist.	Community—pharmacy (individual)	Pharmacist	Baseline	N/A
Pichert et al., 1994 [50]	T1DM	Education	Anchored instruction via “Sydney Meets the Ketone Challenge”	Participants watched a video case study on diabetes sick-day management. Facilitators led group discussion about issues related to the video and solutions were discussed.	Community—camp (group)	Diabetes nurse educator	Education over 2 sessions	N/A
Dye et al., 2022 [48]	T1DM	Education and written information	Sick-day plan	Education was provided to participants after diabetic ketoacidosis admission and sick-day rule plan was provided. Plan was reviewed at every outpatient visit and when emergency line for assistance with blood glucose management was called.	Hospital—outpatient clinic (individual)	N/A	Baseline	N/A
Sedeno et al., 2009 [38]	COPD	Education, written information, ongoing support	Education modules “Living Well with COPD” and action plan	Participants were educated via modules and received a written action plan. A case manager periodically reviewed the participant’s general health and use of self-management strategies.	Community—medical centre (individual)	Case manager, GP	Baseline	N/A
Campain et al., 2023 [34]	COVID, asthma, COPD, CKD, T1DM, T2DM, heart failure, suicide, opioid use	Written information and ongoing support	Sick-day plan	Watch list participants were identified and received a sick-day action plan with regular follow-up from GP teams. GP teams were also notified when these patients entered/were discharged from hospital. Participants were also provided with chronic disease management services, e.g., nursing and allied health assistance, etc., in collaboration with the GP.	Community—medical centre, chronic disease clinic, hospital—inpatient (individual)	GPs, nurses, other allied health	N/A	Individualised
Garrett et al., 1998 [33]	Asthma	Written information	Asthma action plan	Participants received an asthma action plan and adjusted medications according to PEF/symptoms. They were followed up by paediatrician if the exacerbation was not resolved within 3 days.	Hospital—outpatient clinic, inpatient, community—medical centre (individual)	Paediatrician	N/A	Management as per personal PEF
Vitale et al., 2018 [46]	T1DM	Education and written information	Sick-day guideline with fridge magnetic backing	HCPs reviewed the sick-day management guidelines with the participant and were provided with a sick-day plan.	Community—clinic (individual)	Doctor, advanced practice nurse, diabetes educator	Baseline	N/A
Farrell et al., 2019 [51]	T1DM	Phone support service	Mobile phone support	People who experienced sick days contacted the mobile number of the service via a call or text message, to receive advice and take self-action.	Hospital—outpatient clinic (individual)	N/A	N/A	Yes, tailored advice given to patient during contact
Wang et al., 2024 [45]	AKI prevention	Medication review, education and written information	Medication therapy management including action plan	Multidisciplinary care was provided, which included a pharmacist who provided medication therapy management as per the KAMPS framework. This included “kidney function check, advocacy, medication, pressure, and sick-day protocols”.	Hospital—outpatient clinic (individual)	Pharmacist	Baseline, 3 months	N/A
Fireman et al., 1981 [32]	Asthma	Education, phone support service	Individualised education	Management plan was developed based on laboratory studies. The nurse–educator provided education to the patient and family. This was followed by group education with discussion and questions. Patients could contact the nurse via the phone with concerns.	Hospital—outpatient clinic (individual and group)	Nurse educator	Education over 3 sessions	Education tailored to participant/parent’s knowledge
D’Souza et al., 1994 [16]	Asthma	Education, written information, ongoing care	Card-sized self-management plan	Participants attended education clinic and received a self-management plan via GP. They attended a second clinic after 8 weeks. Māori community health workers kept in contact with participants and encouraged them to complete their symptom diaries.	Community—centre (individual)	GP, community health workers	Baseline, 8 weeks	Management as per personal PEF
Charlton et al., 1990 [22]	Asthma	Education, written information, ongoing care	Self-management plan	Participants were randomised to receive a management plan based on symptoms or PEF. They were educated by the practice nurse. Reviewed after 1 week and the plan was altered if necessary. Reviewed every 8 weeks or as often as practice nurse believed appropriate.	Community—clinic (individual)	Practice nurse	Baseline, 1 week, then every 8 weeks	N/A
Yoon et al., 1993 [19]	Asthma	Education and written information	Education program and treatment plan	Five-part education: (1) lecture about asthma, (2) video about asthma treatment, (3) individual training on using a peak flow meter, asthma diaries, and inhaler, (4) a video on typical questions and misconceptions about asthma, and (5) a practical session in using the treatment plan.	Hospital—outpatient clinic (group)	N/A	Baseline	Management as per personal PEF
Cowie et al., 1997 [21]	Asthma	Education and written information	Education session and asthma management plan	Participants were interviewed about their asthma to assess severity of disease, exposure to triggers, etc. Nurse practitioner provided tailored advice before participant was allocated to ‘no action plan’, ‘peak-flow-based action plan’, or ‘symptom-based action plan’ study arms.	Hospital—outpatient clinic (individual)	Nurse practitioner	Baseline	Individualised
Lahdensuo et al., 1996 [29]	Asthma	Education	Patient education and self-adjustment of anti-inflammatory therapy	Participants were educated about asthma via specially trained nurses and learnt breathing and relaxation techniques via physiotherapist. Participants adjusted medications according to daily PEF.	Hospital—outpatient clinic (individual)	Nurse, physiotherapist	Baseline	N/A
Jones et al., 1995 [27]	Asthma	Education and written information	Self-management plan	Participants were provided a self-management plan (symptom-based/PEF-based) and were given education about adjusting medications according to plan. Management plan was revised at 10- and 18-week visits.	Community—medical centre (individual)	GP, practice nurse	5 times: baseline, 2 weeks, 10 weeks, 18 weeks, 26 weeks	Management as per personal PEF
Côté et al., 1998 [18]	Asthma	Education, written information, ongoing care	Individualised education and action plan	Participants attended individualised asthma education program and received an action plan based on PEF. Review by educator every 3 months, who reinforced the usefulness of PEF monitoring.	Hospital—outpatient clinic (individual)	Specialised educator	Baseline, every 3 months	Education was individualised, action plan management as per personal PEF
Beasley et al., 1989 [30]	Asthma	Written information	Self-management plan	Participants provided with self-management action plan. The plan was revised after 2 months of use, if needed.	Hospital—outpatient clinic (individual)	N/A	Baseline, 2 months	Management as per personal PEF
Wong et al., 2017 [28]	Asthma	Education and written information	Pharmaceutical care including Written Asthma Action Plan	Education sessions about asthma, and participants were given an action plan, which was counterchecked by another two pharmacists and two family medicine specialists. Pharmacists also made recommendations for medication changes to physicians.	Community—clinic (individual)	Pharmacist	Baseline, 1 month, 2 months, 3 months, 6 months	Individualised plan
Gallefoss et al., 1999 [24]	Asthma, COPD	Education and written information	Education and self-management Plan	Participants were provided group education about asthma/COPD and received a self-care management plan. They then received individual sessions, where understanding of treatment plan was discussed.	Hospital—outpatient clinic (individual and group)	Nurse, physiotherapist	Baseline	Management as per personal PEF
Van der Palen et al., 1997 [17]	Asthma	Education and written information	Education and personalised written guidelines	Participants were provided with group education and given personalised written guidelines in the last group education session.	Hospital—outpatient clinic (group)	Doctor	Education over 4 sessions	Guidelines personalised as per personal PEF
Bourbeau et al., 2003 [37]	COPD	Education, written information, ongoing support, phone support service	“Living well with COPD” education and action plan	Participants received “Living well with COPD” education at home. They were followed up weekly during the education period and then monthly for the remainder of the study by case managers (HCPs). Participants received a personalised action plan and could contact case managers for advice.	Home (individual)	Case managers (nurse, respiratory therapist, physiotherapist)	Education over 8 weeks	Individualised plan
Sridhar et al., 2008 [36]	COPD	Education, written information, ongoing support	Care package: pulmonary rehabilitation, self-management education, action plan, follow-up	Rehabilitation programme that involved general education and individualised physical training. After the program, a respiratory nurse conducted a home visit and provided a COPD action plan. Nurse followed up with monthly telephone calls and 3-monthly home visits.	Hospital, home (individual)	Respiratory nurse	Education conducted over 4 weeks	Individualised plan
Watson et al., 1997 [35]	COPD	Education and written information	COPD action plan, “A Guide to Living Positively with Chronic Obstructive Pulmonary Disease” education booklet	Practice nurse introduced participants to action plan and education booklet.	Community—medical centre (individual)	Practice nurse	Baseline	Individualised plan
Wood-Baker et al., 2006 [40]	COPD	Education and written information	Education session, COPD written-self management plan, COPD information booklet	Nurse provided education about COPD management, a COPD information booklet, and a card-sized COPD self-management plan.	Community—medical centre (individual)	Respiratory nurse	Baseline	Individualised plan
McGeoch et al., 2006 [39]	COPD	Education and written information	Self-management plan	Participants received standardised self-management education and an individualised self-management plan in additional to usual care.	Community—medical centre (individual)	Practice nurse, respiratory educator	Baseline	Individualised
Herges et al., 2023 [53]	AKI prevention	Medication review, education and written information	Medication review and KAMPS framework implementation	Pharmacists conducted a medication review 30 min before participant visit with their GP, and problems were reviewed with the doctor. Pharmacist reviewed all components of KAMPS framework (kidney function assessment, advocacy/education, medication review, blood pressure assessment, and sick-day counselling).	Community—medical centre (individual)	Pharmacist	Baseline	N/A

AKI = acute kidney injury; CKD = chronic kidney disease; COPD = chronic obstructive pulmonary disorder; GP = general practitioner; HCPs = healthcare professionals; N/A = not available; PEF = peak expiratory flow; T1DM = type 1 diabetes mellitus, T2DM = type 2 diabetes mellitus.

**Table 3:** Summary of guidelines and educational resource characteristics (*n* = 57).

	Number of Documents *n* (%)
Disease state	
Adrenal insufficiency	9 (16%)
Acute kidney injury prevention	3 (5%)
Asthma	14 (25%)
Chronic kidney disease	4 (7%)
Chronic obstructive pulmonary disease	2 (4%)
Heart failure	3 (5%)
Type 1 diabetes	5 (9%)
Type 1 diabetes and type 2 diabetes	8 (14%)
Type 2 diabetes	9 (16%)
Level of organisation (n = 49)	
International	8 (16%)
National	31 (63%)
Local/independent	10 (20%)
Publication year	
Before 2000	3 (5%)
2000–2009	5 (9%)
2010–2019	9 (16%)
2019–2025	26 (46%)
N/A	14 (25%)
Region of origin	
Asia	1 (2%)
Europe	16 (28%)
North America	18 (32%)
Oceania	15 (26%)
International	7 (12%)
Format of patient resource (n = 34)	
Action plan	19 (56%)
Card	1 (3%)
Fact sheet	2 (6%)
Pamphlet	1 (3%)
Patient information sheet	9 (26%)
Website	2 (6%)

## Data Availability

Data is contained within the article or Supplementary Material.

## Supplementary Materials

The following supporting information can be downloaded at https://www.mdpi.com/article/10.3390/medicina61101742/s1.

## Abbreviations

The following abbreviations are used in this manuscript:

AKI	Acute kidney injury
AI	Adrenal insufficiency
CKD	Chronic kidney disease
COPD	Chronic obstructive pulmonary disease
DRPs	Drug-related problems
HCPs	Healthcare professionals
N/A	Not available
PEF	Peak expiratory flow
SDMG	Sick-day medication guidance
T1DM	Type 1 diabetes mellitus
T2DM	Type 2 diabetes mellitus
TIDieR	Template for Intervention Description and Replication
GP	General practice/general practitioner
