# Supplementary material for: Self-Management of Medications During Sick Days for Chronic Conditions: A Scoping Review
Source: Medicina (Kaunas). 2025 Sep 25;61(10):1742. doi: 10.3390/medicina61101742 (PMC12566462; doi:10.3390/medicina61101742)
Supplement: Supplementary file 1 [file medicina-61-01742-s001.zip › medicina-3835435-supplementary.pdf]

Supplementary S1: Preferred Reporting Items for Systematic reviews and Meta-Analyses extension for Scoping Reviews (PRISMA-ScR) Checklist

SECTION	ITEM	PRISMA-ScR CHECKLIST ITEM	REPORTED ON PAGE #
**TITLE**			
Title	1	Identify the report as a scoping review.	1
**ABSTRACT**			
Structured summary	2	Provide a structured summary that includes (as applicable): background, objectives, eligibility criteria, sources of evidence, charting methods, results, and conclusions that relate to the review questions and objectives.	2
**INTRODUCTION**			
Rationale	3	Describe the rationale for the review in the context of what is already known. Explain why the review questions/objectives lend themselves to a scoping review approach.	3
Objectives	4	Provide an explicit statement of the questions and objectives being addressed with reference to their key elements (e.g., population or participants, concepts, and context) or other relevant key elements used to conceptualize the review questions and/or objectives.	3
**METHODS**			
Protocol and registration	5	Indicate whether a review protocol exists; state if and where it can be accessed (e.g., a Web address); and if available, provide registration information, including the registration number.	N/A
Eligibility criteria	6	Specify characteristics of the sources of evidence used as eligibility criteria (e.g., years considered, language, and publication status), and provide a rationale.	4-5
Information sources*	7	Describe all information sources in the search (e.g., databases with dates of coverage and contact with authors to identify additional sources), as well as the date the most recent search was executed.	5
Search	8	Present the full electronic search strategy for at least 1 database, including any limits used, such that it could be repeated.	S2
Selection of sources of evidence†	9	State the process for selecting sources of evidence (i.e., screening and eligibility) included in the scoping review.	5
Data charting process‡	10	Describe the methods of charting data from the included sources of evidence (e.g., calibrated forms or forms that have been tested by the team before their use, and whether data charting was done independently or in duplicate) and any processes for obtaining and confirming data from investigators.	5
Data items	11	List and define all variables for which data were sought and any assumptions and simplifications made.	S3

SECTION	ITEM	PRISMA-ScR CHECKLIST ITEM	REPORTED ON PAGE #
Critical appraisal of individual sources of evidence§	12	If done, provide a rationale for conducting a critical appraisal of included sources of evidence; describe the methods used and how this information was used in any data synthesis (if appropriate).	N/A
Synthesis of results	13	Describe the methods of handling and summarizing the data that were charted.	5
**RESULTS**			
Selection of sources of evidence	14	Give numbers of sources of evidence screened, assessed for eligibility, and included in the review, with reasons for exclusions at each stage, ideally using a flow diagram.	6
Characteristics of sources of evidence	15	For each source of evidence, present characteristics for which data were charted and provide the citations.	10-16
Critical appraisal within sources of evidence	16	If done, present data on critical appraisal of included sources of evidence (see item 12).	N/A
Results of individual sources of evidence	17	For each included source of evidence, present the relevant data that were charted that relate to the review questions and objectives.	13-20
Synthesis of results	18	Summarize and/or present the charting results as they relate to the review questions and objectives.	5-21
**DISCUSSION**			
Summary of evidence	19	Summarize the main results (including an overview of concepts, themes, and types of evidence available), link to the review questions and objectives, and consider the relevance to key groups.	21-24
Limitations	20	Discuss the limitations of the scoping review process.	24
Conclusions	21	Provide a general interpretation of the results with respect to the review questions and objectives, as well as potential implications and/or next steps.	24
**FUNDING**			
Funding	22	Describe sources of funding for the included sources of evidence, as well as sources of funding for the scoping review. Describe the role of the funders of the scoping review.	25

JBI = Joanna Briggs Institute; PRISMA-ScR = Preferred Reporting Items for Systematic reviews and Meta-Analyses extension for Scoping Reviews.
* Where *sources of evidence* (see second footnote) are compiled from, such as bibliographic databases, social media platforms, and Web sites.
† A more inclusive/heterogeneous term used to account for the different types of evidence or data sources (e.g., quantitative and/or qualitative research, expert opinion, and policy documents) that may be eligible in a scoping review as opposed to only studies. This is not to be confused with *information sources* (see first footnote).
‡ The frameworks by Arksey and O'Malley (6) and Levac and colleagues (7) and the JBI guidance (4, 5) refer to the process of data extraction in a scoping review as data charting.

§ The process of systematically examining research evidence to assess its validity, results, and relevance before using it to inform a decision. This term is used for items 12 and 19 instead of "risk of bias" (which is more applicable to systematic reviews of interventions) to include and acknowledge the various sources of evidence that may be used in a scoping review (e.g., quantitative and/or qualitative research, expert opinion, and policy document).

Supplementary S2: Search strategy for respective databases

Embase

#	Searches	Results
1	acute disease/	101928
2	acut* disease*.mp.	106422
3	acut* ill*.mp.	17294
4	acut* unwell.mp.	1112
5	sick-day*.mp.	1149
6	1 or 2 or 3 or 4 or 5	120460
7	drug therapy/	1090219
8	drug therap*.mp.	5716760
9	(medication* or medicine*).mp.	2368088
10	pharmaceutics/	32847
11	drug*.mp.	13807223
12	insulin/	409896
13	long acting insulin/	2537
14	short acting insulin/	2029
15	insulin*.mp.	1029734
16	SADMANS.mp.	3
17	Angiotensin-Converting Enzyme Inhibitors.mp.	19976
18	ACE inhibitor*.mp.	32052
19	ACEi.mp.	11147
20	antihypertensive agent/	121784

21	Angiotensin II Type 1 Receptor Blockers.mp.	480
22	sartans.mp.	713
23	ARB*.mp.	150597
24	diuretic agent/	100286
25	diuretic agent*.mp.	125767
26	diuretic*.mp.	154199
27	nonsteroid antiinflammatory agent/	151147
28	nonsteroid antiinflammatory agent*.mp.	151251
29	NSAIDS.mp.	44098
30	non steroid anti inflamma* agent*.mp.	172
31	sulfonylurea/ or sulfonylurea derivative/	30543
32	sulfonylurea*.mp.	36368
33	sodium glucose cotransporter 2 inhibitor/	16524
34	sodium glucose cotransporter 2 inhibitor*.mp.	18473
35	SGLT2 inhibitor*.mp.	9773
36	SGLT2i.mp.	4074
37	metformin/	92917
38	metformin.mp.	97239
39	glucocorticoid/	112417
40	glucocorticoid.mp.	159239
41	corticosteroid/	314964
42	corticosteroid.mp.	382199
43	inhal* corticosteroid*.mp.	20719
44	digoxin/	50398
45	digoxin.mp.	53357
46	7 or 8 or 9 or 10 or 11 or 12 or 13 or 14 or 15 or 16 or 17 or 18 or 19 or 20 or 21 or 22 or 23 or 24 or 25 or 26 or 27 or 28 or 29 or 30 or 31 or 32 or 33 or 34 or 35 or 36 or 37 or 38 or 39 or 40 or 41 or 42 or 43 or 44 or 45	15853947
47	self care/	80861
48	self care.mp.	91553
49	guidance.mp.	257546
50	recommend*.mp.	1348121
51	protocol*.mp.	985065
52	interven*.mp.	2175874

53	adjust*.mp.	1283767
54	manag*.mp.	3628232
55	discontin*.mp.	284946
56	47 or 48 or 49 or 50 or 51 or 52 or 53 or 54 or 55	8251227
57	(drug therapy/ or drug therap*.mp. or (medication* or medicine*).mp. or pharmaceutics/ or drug*.mp. or insulin/ or long acting insulin/ or short acting insulin/ or insulin*.mp. or SADMANS.mp. or Angiotensin-Converting Enzyme Inhibitors.mp. or ACE inhibitor*.mp. or ACEi.mp. or antihypertensive agent/ or Angiotensin II Type 1 Receptor Blockers.mp. or sartans.mp. or ARB*.mp. or diuretic agent/ or diuretic agent*.mp. or diuretic*.mp. or nonsteroid antiinflammatory agent/ or nonsteroid antiinflammatory agent*.mp. or NSAIDS.mp. or non steroid anti inflamma* agent*.mp. or (sulfonylurea/ or sulfonylurea derivative/) or sulfonylurea*.mp. or sodium glucose cotransporter 2 inhibitor/ or sodium glucose cotransporter 2 inhibitor*.mp. or SGLT2 inhibitor*.mp. or SGLT2i.mp. or metformin/ or metformin.mp. or glucocorticoid/ or glucocorticoid.mp. or corticosteroid/ or corticosteroid.mp. or inhal* corticosteroid*.mp. or digoxin/ or digoxin.mp.) adj20 (self care/ or self care.mp. or guidance.mp. or recommend*.mp. or protocol*.mp. or interven*.mp. or adjust*.mp. or manag*.mp. or discontin*.mp.)	1928146
58	chronic disease/	212789
59	chronic disease*.mp.	288705
60	chronic* ill*.mp.	38379
61	chronic condition*.mp.	45344
62	kidney failure/	168896
63	kidney failure.mp.	447676
64	renal insuffic*.mp.	40598
65	chronic kidney failure/	165411
66	(chronic kidney disease* or chronic kidney failure).mp.	243191
67	(CKD or AKI*).mp. [mp=title, abstract, heading word, drug trade name, original title, device manufacturer, drug manufacturer, device trade name, keyword heading word, floating subheading word, candidate term word]	166478
68	acute kidney injur*.mp.	72739
69	kidney insufficien*.mp.	854
70	cardiovascular disease/	374760
71	cardiovascular disease*.mp.	555229
72	CVD.mp.	85322
73	heart failure/	339311

74	heart failure.mp.	549721
75	adrenal insufficiency/	18406
76	adrenal insufficien*.mp.	21506
77	Addison disease/	7605
78	Addison* disease.mp.	8490
79	endocri* emergenc*.mp.	501
80	adrenal hyperplasia.mp.	16719
81	rheumatoid arthritis/	233694
82	rheumatoid arthriti*.mp.	281484
83	diabetes mellitus/ or diabetic complication/	774655
84	diabetes.mp.	1449690
85	(T1D or T2D).mp. [mp=title, abstract, heading word, drug trade name, original title, device manufacturer, drug manufacturer, device trade name, keyword heading word, floating subheading word, candidate term word]	55257
86	COPD.mp.	118818
87	COPD.mp.	118818
88	chronic obstructive lung disease/	187821
89	asthma/ or allergic asthma/ or exercise induced asthma/	314447
90	asthma.mp.	361706
91	58 or 59 or 60 or 61 or 62 or 63 or 64 or 65 or 66 or 67 or 68 or 69 or 70 or 71 or 72 or 73 or 74 or 75 or 76 or 77 or 78 or 79 or 80 or 81 or 82 or 83 or 84 or 85 or 86 or 87 or 88 or 89 or 90	3666462
92	6 and 57 and 91	3915
93	limit 92 to english language	3595

International Pharmaceutical Abstracts

#	Searches	Results
1	acute disease.mp. [mp=title, subject heading word, registry word, abstract, trade name/generic name]	35
2	acut* illness.mp. [mp=title, subject heading word, registry word, abstract, trade name/generic name]	123
3	acutely unwell.mp. [mp=title, subject heading word, registry word, abstract, trade name/generic name]	1
4	sick day.mp. [mp=title, subject heading word, registry word, abstract, trade name/generic name]	11

5	sick* day.mp. [mp=title, subject heading word, registry word, abstract, trade name/generic name]	11
6	1 or 2 or 3 or 4 or 5	168
7	medication*.mp. [mp=title, subject heading word, registry word, abstract, trade name/generic name]	66032
8	medicin*.mp. [mp=title, subject heading word, registry word, abstract, trade name/generic name]	101114
9	drug*.mp. [mp=title, subject heading word, registry word, abstract, trade name/generic name]	374917
10	insulin*.mp. [mp=title, subject heading word, registry word, abstract, trade name/generic name]	13877
11	Angiotensin-Converting Enzyme Inhibitor*.mp. [mp=title, subject heading word, registry word, abstract, trade name/generic name]	4970
12	ACE inhibitor.mp. [mp=title, subject heading word, registry word, abstract, trade name/generic name]	790
13	ACEi.mp. [mp=title, subject heading word, registry word, abstract, trade name/generic name]	420
14	Angiotensin II Type 1 Receptor Blockers.mp.	10
15	ARBs.mp. [mp=title, subject heading word, registry word, abstract, trade name/generic name]	520
16	sartan*.mp. [mp=title, subject heading word, registry word, abstract, trade name/generic name]	44
17	angiotensin 2.mp. [mp=title, subject heading word, registry word, abstract, trade name/generic name]	29
18	angiotensin 2 receptor blocker*.mp. [mp=title, subject heading word, registry word, abstract, trade name/generic name]	9
19	ARB.mp. [mp=title, subject heading word, registry word, abstract, trade name/generic name]	577
20	diuretic*.mp. [mp=title, subject heading word, registry word, abstract, trade name/generic name]	8572
21	non steroidal anti inflammator*.mp. [mp=title, subject heading word, registry word, abstract, trade name/generic name]	1352
22	NSAIDS.mp. [mp=title, subject heading word, registry word, abstract, trade name/generic name]	2469
23	nonsteroidal anti inflammator*.mp. [mp=title, subject heading word, registry word, abstract, trade name/generic name]	4118
24	nonsteroidal anti-inflammatory drug*.mp. [mp=title, subject heading word, registry word, abstract, trade name/generic name]	2360
25	sulfonylurea*.mp. [mp=title, subject heading word, registry word, abstract, trade name/generic name]	1440
26	Sodium-Glucose Transporter 2 Inhibitors.mp. [mp=title, subject heading word, registry word, abstract, trade name/generic name]	6

27	SGLT2 inhibitor*.mp. [mp=title, subject heading word, registry word, abstract, trade name/generic name]	377
28	SGLT2i*.mp. [mp=title, subject heading word, registry word, abstract, trade name/generic name]	139
29	sodium-glucose cotransporter-2 inhibitor*.mp. [mp=title, subject heading word, registry word, abstract, trade name/generic name]	337
30	SGLT-2 inhibit*.mp. [mp=title, subject heading word, registry word, abstract, trade name/generic name]	119
31	SGLT-2i.mp. [mp=title, subject heading word, registry word, abstract, trade name/generic name]	34
32	Sodium Glucose Co-transporter 2 Inhibitor*.mp. [mp=title, subject heading word, registry word, abstract, trade name/generic name]	153
33	metformin*.mp. [mp=title, subject heading word, registry word, abstract, trade name/generic name]	3998
34	biguanide*.mp. [mp=title, subject heading word, registry word, abstract, trade name/generic name]	316
35	(glibenclamide or chlorpropamide or tolbutamide or glibornuride or tolazamide or carbutamide or glipizide or gliquidone or gliclazide or metahexamide or glisoxepide or glimepiride or acetohexamide).mp. [mp=title, subject heading word, registry word, abstract, trade name/generic name]	2756
36	(captopril or enalapril or lisinopril or perindopril or ramipril or quinapril or benazepril or cilazapril or fosinopril or trandolapril or spirapril or delapril or moexipril or temocapril or zofenopril or imidapril).mp. [mp=title, subject heading word, registry word, abstract, trade name/generic name]	4275
37	(hydrochlorothiazide or chlortalidone or indapamide or furosemide or frusemide or bumetanide or spironolactone or abiraterone).mp. [mp=title, subject heading word, registry word, abstract, trade name/generic name]	5827
38	(losartan or eprosartan or valsartan or irbesartan or tasosartan or candesartan or telmisartan or olmesartan or azilsartan or fimasartan).mp. [mp=title, subject heading word, registry word, abstract, trade name/generic name]	3194
39	(diclofenac or aspirin or celecoxib or etoricoxib or ibuprofen or indometacin or ketoprofen or ketorolac or mefenamic acid or meloxicam or naproxen or parecoxib or piroxicam).mp. [mp=title, subject heading word, registry word, abstract, trade name/generic name]	20208
40	(dapagliflozin or canagliflozin or empagliflozin or ertugliflozin or ipragliflozin or sotagliflozin or luseogliflozin or bexagliflozin).mp. [mp=title, subject heading word, registry word, abstract, trade name/generic name]	953

41	glucocorticoid*.mp. [mp=title, subject heading word, registry word, abstract, trade name/generic name]	2058
42	corticosteroid*.mp. [mp=title, subject heading word, registry word, abstract, trade name/generic name]	8466
43	(prednisone or prednisolone or betamethasone or dexamethasone or fluocortolone or methylprednisolone or paramethasone or triamcinolone or hydrocortisone or cortisone or prednylidene or rimexolone or deflazacort or cloprednol or meprednisone or cortivazol or vamorolone).mp. [mp=title, subject heading word, registry word, abstract, trade name/generic name]	14784
44	digoxin.mp. [mp=title, subject heading word, registry word, abstract, trade name/generic name]	2971
45	7 or 8 or 9 or 10 or 11 or 12 or 13 or 14 or 15 or 16 or 17 or 18 or 19 or 20 or 21 or 22 or 23 or 24 or 25 or 26 or 27 or 28 or 29 or 30 or 31 or 32 or 33 or 34 or 35 or 36 or 37 or 38 or 39 or 40 or 41 or 42 or 43 or 44	489334
46	self care.mp. [mp=title, subject heading word, registry word, abstract, trade name/generic name]	873
47	asthma action plan.mp. [mp=title, subject heading word, registry word, abstract, trade name/generic name]	16
48	action plan.mp. [mp=title, subject heading word, registry word, abstract, trade name/generic name]	306
49	guidance.mp. [mp=title, subject heading word, registry word, abstract, trade name/generic name]	3624
50	guide.mp. [mp=title, subject heading word, registry word, abstract, trade name/generic name]	5276
51	recommend*.mp. [mp=title, subject heading word, registry word, abstract, trade name/generic name]	33651
52	protocol.mp. [mp=title, subject heading word, registry word, abstract, trade name/generic name]	7267
53	interven*.mp. [mp=title, subject heading word, registry word, abstract, trade name/generic name]	56977
54	adjust*.mp. [mp=title, subject heading word, registry word, abstract, trade name/generic name]	22801
55	manag*.mp. [mp=title, subject heading word, registry word, abstract, trade name/generic name]	62661
56	discontin*.mp. [mp=title, subject heading word, registry word, abstract, trade name/generic name]	15508
57	46 or 48 or 49 or 50 or 51 or 52 or 53 or 54 or 55 or 56	164994
58	((medication* or medicin* or drug* or insulin* or Angiotensin-Converting Enzyme Inhibitor* or ACE inhibitor or ACEi or Angiotensin II Type 1 Receptor Blockers or ARBs or sartan* or angiotensin 2 or angiotensin 2 receptor blocker* or ARB or diuretic* or non steroidal anti inflammator* or NSAIDS or nonsteroidal anti inflammator* or nonsteroidal anti-inflammatory drug* or sulfonylurea* or Sodium-Glucose Transporter 2 Inhibitors or SGLT2 inhibitor* or SGLT2i* or sodium-glucose cotransporter-2 inhibitor* or SGLT-2 inhibit* or SGLT-2i or	54814

	Sodium Glucose Co-transporter 2 Inhibitor* or metformin* or biguanide* or (glibenclamide or chlorpropamide or tolbutamide or glibornuride or tolazamide or carbutamide or glipizide or gliquidone or gliclazide or metahexamide or glisoxepide or glimepiride or acetohexamide) or (captopril or enalapril or lisinopril or perindopril or ramipril or quinapril or benazepril or cilazapril or fosinopril or trandolapril or spirapril or delapril or moexipril or temocapril or zofenopril or imidapril) or (hydrochlorothiazide or chlortalidone or indapamide or furosemide or frusemide or bumetanide or spironolactone or abiraterone) or (losartan or eprosartan or valsartan or irbesartan or tasosartan or candesartan or telmisartan or olmesartan or azilsartan or fimasartan) or (diclofenac or aspirin or celecoxib or etoricoxib or ibuprofen or indometacin or ketoprofen or ketorolac or mefenamic acid or meloxicam or naproxen or parecoxib or piroxicam) or (dapagliflozin or canagliflozin or empagliflozin or ertugliflozin or ipragliflozin or sotagliflozin or luseogliflozin or bexagliflozin) or glucocorticoid* or corticosteroid* or (prednisone or prednisolone or betamethasone or dexamethasone or fluocortolone or methylprednisolone or paramethasone or triamcinolone or hydrocortisone or cortisone or prednylidene or rimexolone or deflazacort or cloprednol or meprednisone or cortivazol or vamorolone) or digoxin) adj20 (self care or action plan or guidance or guide or recommend* or protocol or interven* or adjust* or manag* or discontin*)).mp.	
59	chronic disease*.mp. [mp=title, subject heading word, registry word, abstract, trade name/generic name]	6387
60	chronic condition*.mp. [mp=title, subject heading word, registry word, abstract, trade name/generic name]	779
61	multiple chronic condition*.mp. [mp=title, subject heading word, registry word, abstract, trade name/generic name]	51
62	chronic obstructive pulmonary disease.mp. [mp=title, subject heading word, registry word, abstract, trade name/generic name]	2140
63	COPD.mp. [mp=title, subject heading word, registry word, abstract, trade name/generic name]	1784
64	renal insuff*.mp. [mp=title, subject heading word, registry word, abstract, trade name/generic name]	1237
65	kidney failure*.mp. [mp=title, subject heading word, registry word, abstract, trade name/generic name]	2813
66	chronic kidney disease.mp. [mp=title, subject heading word, registry word, abstract, trade name/generic name]	1765
67	kidney disease*.mp. [mp=title, subject heading word, registry word, abstract, trade name/generic name]	6039
68	acute kidney injur*.mp. [mp=title, subject heading word, registry word, abstract, trade name/generic name]	853
69	AKI.mp. [mp=title, subject heading word, registry word, abstract, trade name/generic name]	399

70	kidney insuff*.mp. [mp=title, subject heading word, registry word, abstract, trade name/generic name]	15
71	cardiovascular disease*.mp. [mp=title, subject heading word, registry word, abstract, trade name/generic name]	11009
72	CVD.mp. [mp=title, subject heading word, registry word, abstract, trade name/generic name]	952
73	heart failure.mp. [mp=title, subject heading word, registry word, abstract, trade name/generic name]	8256
74	(hfpef or hfref).mp. [mp=title, subject heading word, registry word, abstract, trade name/generic name]	255
75	adrenal insuffi*.mp. [mp=title, subject heading word, registry word, abstract, trade name/generic name]	163
76	addison* disease*.mp. [mp=title, subject heading word, registry word, abstract, trade name/generic name]	41
77	addison's disease.mp. [mp=title, subject heading word, registry word, abstract, trade name/generic name]	39
78	hypoadrenocorticism.mp. [mp=title, subject heading word, registry word, abstract, trade name/generic name]	2
79	hypoaldosteronism.mp. [mp=title, subject heading word, registry word, abstract, trade name/generic name]	18
80	endocri* emergenc*.mp. [mp=title, subject heading word, registry word, abstract, trade name/generic name]	5
81	adrenal hyperplasia.mp. [mp=title, subject heading word, registry word, abstract, trade name/generic name]	55
82	hyperandrogenism.mp. [mp=title, subject heading word, registry word, abstract, trade name/generic name]	80
83	adrenal hyperplasia.mp. [mp=title, subject heading word, registry word, abstract, trade name/generic name]	55
84	rheumatoid arthritis.mp. [mp=title, subject heading word, registry word, abstract, trade name/generic name]	6789
85	rheumatic arthritis.mp. [mp=title, subject heading word, registry word, abstract, trade name/generic name]	44
86	diabetes mellitus.mp. [mp=title, subject heading word, registry word, abstract, trade name/generic name]	19535
87	(t1d or t2d).mp. [mp=title, subject heading word, registry word, abstract, trade name/generic name]	613

88	(type 1 diabetes or type 2 diabetes).mp. [mp=title, subject heading word, registry word, abstract, trade name/generic name]	8555
89	t2dm.mp. [mp=title, subject heading word, registry word, abstract, trade name/generic name]	1270
90	asthma.mp. [mp=title, subject heading word, registry word, abstract, trade name/generic name]	8742
91	59 or 60 or 61 or 62 or 63 or 64 or 65 or 66 or 67 or 68 or 69 or 70 or 71 or 72 or 73 or 74 or 75 or 76 or 77 or 78 or 79 or 80 or 81 or 82 or 83 or 84 or 85 or 86 or 87 or 88 or 89 or 90	65553
92	6 and 58 and 91	18
93	limit 92 to english language	16
94	(harm adj5 redu*).mp. [mp=title, subject heading word, registry word, abstract, trade name/generic name]	355
95	unwell.mp. [mp=title, subject heading word, registry word, abstract, trade name/generic name]	34
96	illness.mp. [mp=title, subject heading word, registry word, abstract, trade name/generic name]	7296
97	1 or 2 or 3 or 4 or 5 or 95 or 96	7368
98	58 and 91 and 97	225
99	limit 98 to english language	212

Medline

#	Searches	Results
1	Acute Disease/	225850
2	acute disease.mp.	228827
3	acut* illness.mp.	5462
4	acutely unwell.mp.	507
5	sick day.mp.	194
6	sick* day.mp.	197
7	1 or 2 or 3 or 4 or 5 or 6	233842
8	medication.mp.	323842
9	medicine.mp.	1058822
10	Pharmaceutical Preparations/	70218
11	pharmaceutical preparations.mp.	73461
12	drug*.mp.	6844873
13	insulins/ or biphasic insulins/ or insulin, long-acting/ or insulin detemir/ or insulin glargine/ or insulin, isophane/ or insulin, lente/ or insulin, ultralente/ or insulin, short-acting/ or insulin/ or insulin, regular, human/ or isophane insulin, human/ or insulin, regular, pork/	206744
14	insulin.mp.	481102

15	SADMANS.mp.	3
16	Angiotensin-Converting Enzyme Inhibitors/	36576
17	angiotensin-converting enzyme inhibitors.mp.	43461
18	ACE inhibitor*.mp.	19481
19	ACEi.mp.	5234
20	Angiotensin II Type 1 Receptor Blockers/	9127
21	Angiotensin II Type 1 Receptor Blockers.mp.	9303
22	sartans.mp.	345
23	ARB*.mp.	129971
24	Diuretics/	29883
25	diuretic*.mp.	59601
26	Anti-Inflammatory Agents, Non-Steroidal/	73067
27	non steroidal anti inflammator*.mp.	20229
28	NSAIDS.mp.	24245
29	Sulfonylurea Compounds/	6778
30	sulfonylurea*.mp.	13354
31	Sodium-Glucose Transporter 2 Inhibitors/	6772
32	Sodium-Glucose Transporter 2 Inhibitors.mp.	6961
33	SGLT2 inhibitor*.mp.	5921
34	SGLT2i.mp.	2077
35	Sodium Glucose Co-transporter 2 Inhibitor*.mp.	1763
36	metformin/ or sitagliptin phosphate, metformin hydrochloride drug combination/	18844
37	metformin.mp.	31935
38	metformin hydrochloride drug combination.mp.	8
39	adrenal cortex hormones/ or glucocorticoids/	141643
40	glucocortcoids.mp.	10
41	corticosteroids.mp.	85590
42	Digoxin/	11460
43	digoxin.mp.	15985
44	8 or 9 or 10 or 11 or 12 or 13 or 14 or 15 or 16 or 17 or 18 or 19 or 20 or 21 or 22 or 23 or 24 or 25 or 26 or 27 or 28 or 29 or 30 or 31 or 32 or 33 or 34 or 35 or 36 or 37 or 38 or 39 or 40 or 41 or 42 or 43	8359774
45	Self Care/	36637
46	self care.mp.	53912

47	action plan.mp.	6434
48	guidance.mp.	179389
49	guide.mp.	277635
50	recommend*.mp.	913585
51	protocol.mp.	471501
52	interven*.mp.	1549114
53	adjust*.mp.	901677
54	manag*.mp.	2049640
55	discontin*.mp.	160787
56	45 or 46 or 47 or 48 or 49 or 50 or 51 or 52 or 53 or 54 or 55	5442333
57	((medication or medicine).mp. or Pharmaceutical Preparations/ or pharmaceutical preparations.mp. or drug*.mp. or (insulins/ or biphasic insulins/ or insulin, long-acting/ or insulin detemir/ or insulin glargine/ or insulin, isophane/ or insulin, lente/ or insulin, ultralente/ or insulin, short-acting/ or insulin/ or insulin, regular, human/ or isophane insulin, human/ or insulin, regular, pork/) or insulin.mp. or SADMANS.mp. or Angiotensin-Converting Enzyme Inhibitors/ or angiotensin-converting enzyme inhibitors.mp. or ACE inhibitor*.mp. or ACEi.mp. or Angiotensin II Type 1 Receptor Blockers/ or Angiotensin II Type 1 Receptor Blockers.mp. or sartans.mp. or ARB*.mp. or Diuretics/ or diuretic*.mp. or Anti-Inflammatory Agents, Non-Steroidal/ or non steroidal anti inflammator*.mp. or NSAIDS.mp. or Sulfonylurea Compounds/ or sulfonylurea*.mp. or Sodium-Glucose Transporter 2 Inhibitors/ or Sodium-Glucose Transporter 2 Inhibitors.mp. or SGLT2 inhibitor*.mp. or SGLT2i.mp. or Sodium Glucose Co-transporter 2 Inhibitor*.mp. or (metformin/ or sitagliptin phosphate, metformin hydrochloride drug combination/) or metformin.mp. or metformin hydrochloride drug combination.mp. or (adrenal cortex hormones/ or glucocorticoids/) or glucocortcoids.mp. or corticosteroids.mp. or Digoxin/ or digoxin.mp.) adj20 (Self Care/ or self care.mp. or action plan.mp. or guidance.mp. or guide.mp. or recommend*.mp. or protocol.mp. or interven*.mp. or adjust*.mp. or manag*.mp. or discontin*.mp.)	682901
58	chronic disease/ or "chronic kidney disease-mineral and bone disorder"/ or multiple chronic conditions/ or pulmonary disease, chronic obstructive/ or renal insufficiency, chronic/	380375
59	chronic disease*.mp.	359821
60	chronic condition*.mp.	28026
61	renal insufficiency, chronic/ or "chronic kidney diseases of uncertain etiology"/ or kidney failure, chronic/	139363
62	chronic kidney disease.mp.	82997
63	CKD.mp.	48088

64	renal insufficiency/ or acute kidney injury/ or kidney tubular necrosis, acute/ or cardio-renal syndrome/ or renal insufficiency, chronic/ or "chronic kidney diseases of uncertain etiology"/ or kidney failure, chronic/	210562
65	acute kidney injury.mp.	76103
66	AKI.mp.	24080
67	kidney insufficien*.mp.	817
68	cardiovascular diseases/ or heart diseases/ or vascular diseases/	297518
69	cardiovascular disease.mp.	183377
70	CVD.mp.	55200
71	heart failure/ or cardio-renal syndrome/ or heart failure, diastolic/ or heart failure, systolic/	153716
72	heart failure.mp.	269574
73	adrenal insufficiency/ or addison disease/ or hypoadrenocorticism, familial/ or hypoaldosteronism/	11548
74	addison* disease.mp.	6079
75	endocri* emergenc*.mp.	291
76	adrenal hyperplasia, congenital/ or hyperandrogenism/	9788
77	adrenal hyperplasia.mp.	10412
78	Arthritis, Rheumatoid/	112883
79	rheumatoid arthrit*.mp.	126963
80	Diabetes Mellitus, Type 1/ or Diabetes Mellitus/ or Diabetes Complications/ or Diabetes Mellitus, Type 2/	416790
81	diabetes.mp.	799405
82	(T1D or T2D).mp.	29048
83	COPD.mp.	62525
84	asthma/ or asthma, aspirin-induced/ or asthma, exercise-induced/ or asthma, occupational/ or asthma-chronic obstructive pulmonary disease overlap syndrome/ or status asthmaticus/	145900
85	asthma.mp.	205055
86	58 or 59 or 60 or 61 or 62 or 63 or 64 or 65 or 66 or 67 or 68 or 69 or 70 or 71 or 72 or 73 or 74 or 75 or 76 or 77 or 78 or 79 or 80 or 81 or 82 or 83 or 84 or 85	2305472
87	7 and 57 and 86	2496
88	limit 87 to english language	2254

SCOPUS
TITLE-ABS-KEY "action plan" OR "sick day" OR "acute illness" OR "acutely unwell" OR "sick day rule" OR "sick day action plan" OR "sick day medication guid*" OR "sick day protocol" OR "sick day manag*"

Google Scholar Search
"action plan" OR "sick day" OR "acute illness" OR acutely AND unwell OR "sick day rule" OR "sick day action plan" OR "sick day medication guid*" OR "sick-day protocol" OR "sick-day manag*"

Supplementary S3: Definitions of data items for data extraction table

Item	Definition
Author(s), Year	List of authors by surname, year of publication.
Condition	Condition that the intervention addresses.
Intervention type	Education: transfer of knowledge and skills through teaching or training.<br>Written information: any information documented in a readable format.<br>Ongoing support: regular close contact with participant to maintain progress, address issues and answer questions. Does not include follow ups to collect outcome data.<br>Phone support service: phone service that participants can use to contact support.<br>Medication review: evaluation of participant's medications with the aim of optimisation of therapy.
Intervention name	Name of intervention
Intervention description	Description of intervention provided to participant.
Setting (format)	Location in which intervention was provided including hospital (inpatient clinic, outpatient clinic), community (medical centre, pharmacy, camp, chronic disease clinic, community centre, clinic), home.<br>Mode of delivery of intervention: individual, group setting.
Facilitator	Type of healthcare professional that provided the intervention e.g. nurse, general practitioner, pharmacists, physiotherapist, other.
How often intervention was delivered	How often the intervention was delivered to participant.
Tailoring to participant	How the intervention was altered to the participant as described by the study.
